# Supplementary material for: Symbolic recording of signalling and cis-regulatory element activity to DNA
Source: Nature. 2024 Jul 17;632(8027):1073–81. doi: 10.1038/s41586-024-07706-4 (PMC11357993; doi:10.1038/s41586-024-07706-4)
Supplement: Supplementary file 1 — This file contains Supplementary Notes 1 and 2, Figs. 1 and 2 and references. [file 41586_2024_7706_MOESM1_ESM.pdf]

nature portfolio

https://doi.org/10.1038/s41586-024-07706-4

Supplementary information

Symbolic recording of signalling and *cis*-regulatory element activity to DNA

In the format provided by the authors and unedited

SUPPLEMENTARY INFORMATION

Contents:

Supplementary Note 1: Early development and optimization of ENGRAM

Supplementary Figure 1: Early development and optimization of ENGRAM

Supplementary Note 2: Attempts to record endogenous transcription with ENGRAM

Supplementary Figure 2: Attempts to record endogenous transcription with ENGRAM

References

Supplementary Note 1. Early development and optimization of ENGRAM

Early tests of the ENGRAM recording strategy

We first developed 3' ENGRAM v0, in which *csy4*-pegRNA-*csy4* was embedded within the 3' untranslated region (UTR) of a CRE-minP-driven GFP transcript (**Supplementary Figure 1a**). When coupled to a constitutive Pol-2 PGK promoter and introduced into a HEK293T cell line constitutively expressing both Csy4 and a prime editor (PE2)[1], 3' ENGRAM v0 exhibited comparable editing efficiency to traditional U6-driven pegRNAs (5.9% v.s. 5.3%; **Supplementary Figure 1b**). However, a comparison of active CREs vs. a minimal promoter alone found only a modest difference (2.1-fold; **Supplementary Figure 1c**), possibly due to the accumulation of background edits during transfection of the constitutively Csy4-expressing cell line. To reduce such background, we designed 3' ENGRAM, in which the GFP is replaced by Csy4 in the recorder, such that the system no longer relies on constitutively expressed Csy4 (**Supplementary Figure 1d**). This change resulted in a 2.9-fold reduction in background editing (1.4% → 0.5% at 3 days post-transfection; **Supplementary Figure 1e**).

Speculation on the cause of background accumulation

In the main text, we describe variants of ENGRAM in which the *csy4* hairpin-flanked pegRNA is differentially positioned. For all versions tested, background editing plateaued after several days, suggesting that it primarily accumulates shortly after transfection (**Figure 1c**). We speculate that this background editing is due to bacterial origin-of-replication-driven transcription on plasmids[2] prior to integration, rather than CRE/minP-driven transcription from integrated recorders.

Optimizing ENGRAM in terms of the guide (pegRNA vs. epegRNA), prime editor (PE2 vs. PEmax), or pegRNA release strategy (*csy4* hairpins vs. tRNA)

While ENGRAM was under development, studies showed that engineered pegRNAs (epegRNAs; modified with tevoPreQ1 hairpin)[3] or optimized prime editors (PEmax)[4] can improve prime editing efficiency. To test epegRNAs in the context of ENGRAM, we transiently transfected PE2(+) HEK293T cells with constitutively active ENGRAM recorders bearing either pegRNAs or epegRNAs encoding a degenerate 5-mer insertion (5N). Surprisingly, at 3 days post-transfection, we observed a lower editing efficiency with epegRNAs than pegRNAs (17% vs 22%, respectively; **Supplementary Figure 1f**). We speculate that the *csy4* hairpin might already

serve a similar role to *tevoPreQ1* in protecting pegRNAs from degradation and/or that the presence of both the *tevoPreQ1* and *csy4* hairpins might disrupt RNA folding.

We initially tested PE2 vs. PEmax[4] in K562 cells. In this experiment, constitutively active ENGRAM recorders bearing 5N insertions, together with PE2 or PEmax constructs, were transiently transfected in triplicate. Cells were harvested 3 days post-transfection, and insertions at the endogenous *HEK3* locus quantified. We observed a 1.7-fold greater editing efficiency with PEmax compared to PE2 (**Supplementary Figure 1g**), consistent with PEmax's original description[1].

In separate experiments conducted later in the project, we tested PE2 vs. PEmax in mouse embryonic stem cells (mESCs). In contrast with the previous experiment, we first leveraged the piggyBac system to genomically integrate both a synthetic *HEK3* target site to serve as DNA Tape (because the endogenous human *HEK3* site is not present in the mouse genome) as well as constructs bearing constitutively expressed PE2 or PEmax. To the resulting polyclonal mESC cell lines, we transiently transfected constitutively active ENGRAM recorders bearing 5N insertions. Cells were harvested 3 days post-transfection, and insertions at synthetic *HEK3* target sites quantified. We observed a 9.3-fold increase in the editing efficiency with PEmax over PE2 in these mESCs, substantially higher than the 1.7-fold difference observed for PEmax vs. PE2 in K562s (**Supplementary Figure 1g**). However, because there are additional contrasts between these experiments (*e.g.* transient vs. integrated prime editor; endogenous vs. synthetic *HEK3* site; human vs. mouse translational machinery intersecting with non-codon-optimized [PE2] or human-codon-optimized [PEmax] RT domain), we cannot draw clear conclusions about which factor(s) are explanatory.

We also tested whether tRNAs[5] could be used as an alternative to *csy4* to facilitate pegRNA release. However, upon replacing the *csy4* hairpins with tRNA sequences, we observed a nearly complete ablation of recording activity (**Supplementary Figure 1h**). Overall, these experiments suggest that future ENGRAM experiments would benefit from switching to more active prime editors such as PEmax, but not from switching to epegRNAs nor tRNAs.

<u>The ENGRAM recorder system does not substantially alter the cellular transcriptome</u>

To assess whether ENGRAM components impact cell state, we compared the bulk transcriptomes of HEK293T cells that were unmodified, modified with integrated PE2, or modified with both integrated PE2 and the ENGRAM NFκB recorder, harvesting +/- TNFα stimulation. About half (52%) of the observed transcriptional variation was associated with the derivation of a PE2 monoclonal line, and an additional 34% associated with TNFα treatment (**Supplementary Figure 1i**). Of note, there were no significantly differentially expressed genes in PE2(+), TNFα-treated cells that did vs. did not have an integrated ENGRAM NFκB recorder (**Supplementary Figure 1j**). In other words, the presence of the ENGRAM recorder, even when activated by TNFα, did not appear to substantially impact the transcriptome above and beyond changes attributable to other variables.

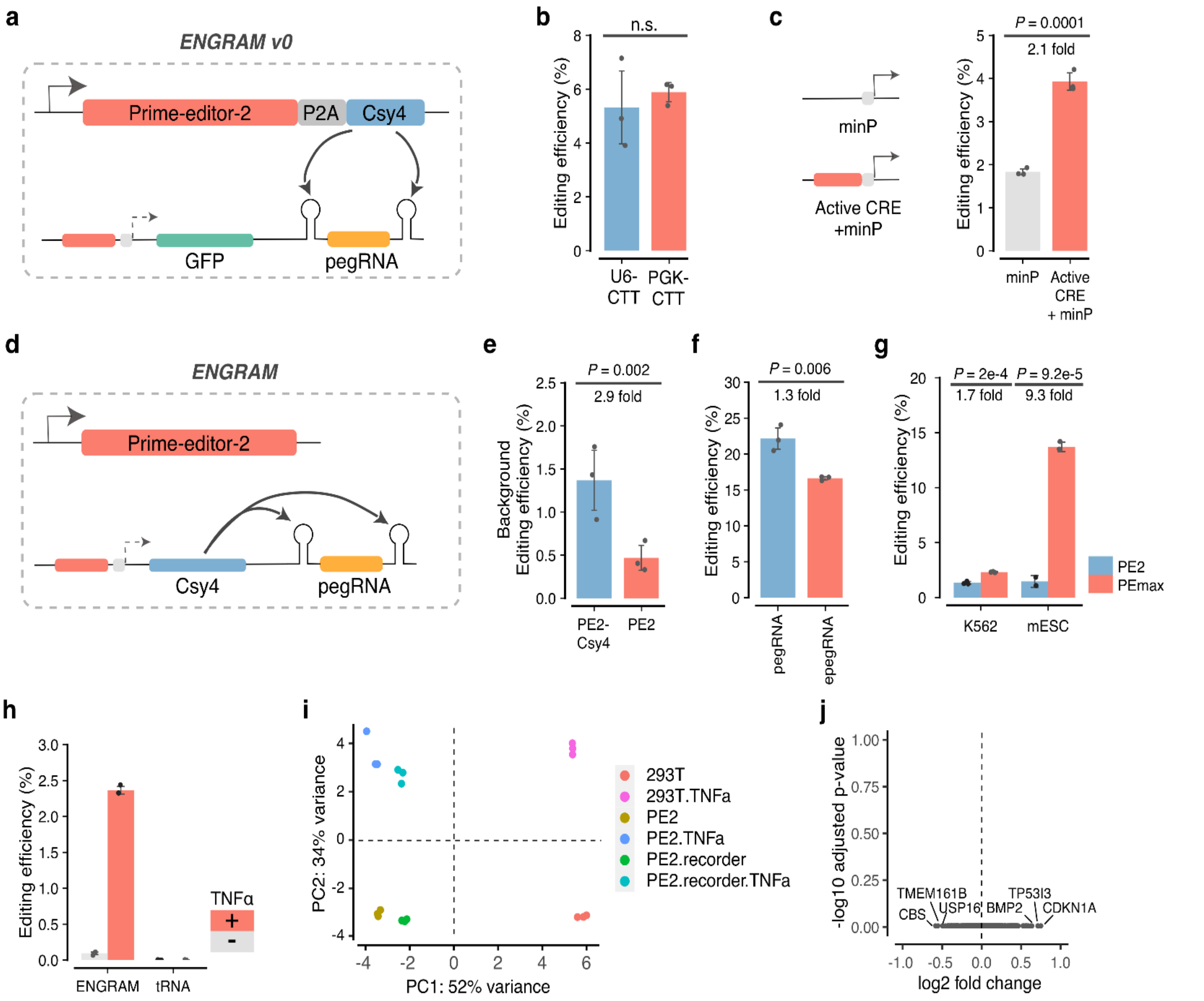

Supplementary Figure 1. Early development and optimization of ENGRAM. (a) Schematic of ENGRAM v0 recorder. A pegRNA writing unit is flanked by *csy4* hairpins and embedded within the 3' UTR of a Pol-2-driven GFP mRNA. PE2 and Csy4 are constitutively expressed from a separate locus. Csy4 cleaves at the *csy4* hairpins and releases the active pegRNA. **(b)** Across three transfection replicates, the ENGRAM v0 recorder driven by a constitutive Pol-2 PGK promoter (PGK-CTT) exhibited comparable efficiency for inserting CTT at the *HEK3* locus to a U6-driven CTT-pegRNA (U6-CTT). In the K562 cell line in which this experiment was performed, PE2 and Csy4 were constitutively expressed. **(c)** A schematic of the constructs used for two pools of ENGRAM v0 recorders is shown on the left, and the observed editing efficiency for each pool on the right. Briefly, a pool of 13 CREs known to be active in this cell line[6], cloned upstream of minP and driving a pool of pegRNAs encoding insertion of a 5N degenerate sequence to *HEK3*, was 2.1-fold more active than a similar library of control constructs bearing minP alone. Error bars correspond to standard deviations across 3 transfection replicates. P-values were obtained using the two-tailed Student's t-test. **(d)** Schematic of ENGRAM 2.0 recorder. A pegRNA writing unit is flanked by *csy4* hairpins and embedded within the 3' or 5' UTR of a Pol-2-driven Csy4 mRNA. PE2 (or PEmax) is constitutively expressed from a separate locus. When the ENGRAM recorder is active, Csy4 is produced,

cleaves at the *csy4* hairpins and releases the active pegRNA. **(e)** The ENGRAM v0 and ENGRAM recorder libraries (constitutively active and programming a degenerate 5N insertion to the *HEK3* locus) were separately co-transfected with plasmids encoding PE2-Csy4 and PE2, respectively. ENGRAM 2.0 exhibited 2.9-fold less background recording than ENGRAM v0. Error bars correspond to standard deviations across 3 transfection replicates. **(f)** Comparison of recording efficiency between epegRNAs vs. pegRNAs. Libraries of epegRNAs or pegRNAs (constitutively active and programming a degenerate 5N insertion to the *HEK3* locus) were cloned into the 5' ENGRAM 2.0 architecture. These two libraries were transiently transfected, separately, into PE2(+) HEK293T cells in triplicate. Genomic DNA was harvested 3 days post-transfection. Unexpectedly, pegRNAs showed 30% higher recording efficiency than epegRNAs. We speculate that in ENGRAM, the *csy4* hairpin might already serve to protect pegRNAs from degradation, and the double hairpin at the end of pegRNA might affect the RNA folding, reducing efficiency. Error bars correspond to standard deviations across 3 transfection replicates. P-values were obtained using the two-tailed Student's t-test. **(g)** Comparison of recording efficiency between prime editor variants. PE2 or PEmax, together with a library of constitutive ENGRAM recorders programming a degenerate 5N insertion to the *HEK3* locus, were co-transfected into K562 cells. In a separate experiment, an ENGRAM recorder encoding a 5-bp insertion was transiently transfected into mESCs bearing a synthetic DNA Tape (*HEK3*) and stably expressing PE2 or PEmax. Genomic DNA was harvested 3 days post-transfection and insertions at endogenous (K562) or synthetic (mESC) *HEK3* loci were quantified. We observed that PEmax drove 1.7-fold and 9.3-fold more efficient recording than PE2 in K562 cells and mESCs, respectively. Although we used PE2 for many of the experiments reported in this paper (as PEmax had yet to be described at the time that they were performed), we recommend using PEmax for all future recording assays. Error bars correspond to standard deviations across 3 transfection replicates. P-values were obtained using the two-tailed Student's t-test. **(h)** tRNA processing for pegRNA release is not compatible with ENGRAM. We replaced *csy4* hairpin with tRNA sequences as an alternative approach for pegRNA release. Pol-2 transcripts bearing pegRNAs flanked by either *csy4* hairpins or tRNA sequences, in both cases encoding degenerate 5N insertions, were driven by the NFκB response element. Recorders were integrated to the genome via piggyBac, and their activities were measured in the absence or presence of 10ng/ml TNFα in triplicate. In contrast with *csy4* hairpin-flanked pegRNAs, tRNA-flanked pegRNAs failed to exhibit recording activity in the presence of TNFα. **(i, j)** Measuring the effects of ENGRAM components on cell functions using bulk RNA sequencing. We profiled bulk RNA expression in HEK293T cells, either with no integrated modifications (293T), with integrated PE2 (PE2, monoclonal line), or with integrated PE2 and ENGRAM NFκB recorder (PE2.recorder, derived from PE2 line mentioned above), harvesting either without vs. after TNFα stimulation. **(i)** PCA plot of bulk RNA-seq data from various conditions. About half (52%) of the observed transcriptional variation was associated with derivation of the PE2 monoclonal line, and an additional 34% associated with TNFα treatment. **(j)** Volcano plot showing differentially expressed genes between the PE2+TNFα and PE2.recorder+TNFα conditions. No significant changes in gene expression were detected (Wald-test with Benjamini-Hochberg correction $P < 0.05$ for a change >50% ($\log_2$-fold change >0.58)).

Supplementary Note 2. Attempts to record endogenous transcription with ENGRAM

As a large portion of the mammalian genome is transcribed[7], we reasoned that random integration of the "minimal" portion of the ENGRAM cassette containing the hairpin-flanked pegRNA might enable the global recording of transcriptional activity across the entire genome. To test this idea, we devised a strategy to capture both the integration site of minimal ENGRAM cassettes (*csy4*-pegRNA-*csy4*, flanked by a T7 promoter, and a unique barcode) and their transcriptional activities. In this setup, there is no enhancer or minimal promoter (**Supplementary Figure 2a**). Rather the idea is that many of these minimal ENGRAM cassettes would be integrated into introns or UTRs, their pegRNAs transcribed as part of hnRNA, released by Csy4, and then free to write specific insertions to the endogenous *HEK3* locus (DNA Tape) at a rate proportional to their abundance.

After bottlenecking a population of cells in which this construct had been randomly integrated, we used a T7 *in vitro* transcription assay to map the integration site of every pegRNA construct[8], while also identifying which unique insertional barcode was associated with which unique integration site (**Supplementary Figure 2a**). Then, after expressing PE2 and Csy4 in these cells, we sequenced barcodes that had been written to the endogenous *HEK3* DNA Tape, and sought to ask whether recording levels correlated with endogenous transcription levels of the genes to which they were integrated. Overall, we mapped 109,626 integration sites across 10 pools of cells (~1000 cells per pool, median 10,730 integration sites per pool as we were at an MOI of ~10) and classified them based on their site of integration. Broadly they fell into two subgroups: genic (promoter, exon, and intron) and intergenic (long/short non-coding RNA, ribosomal RNAs and unannotated regions). Recording efficiencies of the endogenous *HEK3* site were modest (1-2%), but sufficient for reasonable reproducibility (**Supplementary Figure 2b-d**). Of note, the same population of cells expressing PE2 alone did not show detectable recording activities, suggesting the observed recording is truly driven by the minimal ENGRAM cassettes integrated into diverse sites across the genome (**Supplementary Figure 2d**).

However, the results were different from what we anticipated. Among the insertional barcodes written to the DNA Tape, ~77% were associated with the 0.33% of pegRNA cassettes whose integration sites fell within the 45S ribosomal DNA array (rDNA), a stunning 238-fold enrichment. The 45S rDNA array consists of repeats of a 45 kb rDNA unit, including an actively transcribed region (from 5' ETS to 3' ETS) and an intergenic spacer (**Supplementary Figure 2i**). Within each rDNA unit, almost all active ENGRAM cassettes (*i.e.* those for which the corresponding barcode was written to DNA Tape) mapped to the Crick strand of the active transcription unit (**Supplementary Figure 2i-j**). Thus, we can attribute the majority of recording via "minimal ENGRAM cassettes" that happened to integrate within rDNA to the highly active transcription of ribosomal genes.

In contrast, we did not observe appreciable differences between other classes of integration sites, nor did we observe the expected correlation between recording levels from pegRNAs integrated within Pol-2-transcribed genes and their endogenous expression levels (**Supplementary Figure 2h,k**). This is not what we expected, and we still do not fully understand this result. However, we speculate that recording of endogenous transcription may be modulated by many more factors than is the case when ENGRAM recorders are placed within a more constant context. This includes the extent to which heterogeneous nuclear RNA (hnRNA) is accessible to Csy4 processing, hnRNA pre-processing, etc. Such factors might allow us to capture a massive enrichment for rRNAs that happened to land within highly transcribed rRNAs, while still leaving us unable to quantitatively record from more lowly expressed Pol-2 transcripts in which the minimal ENGRAM cassette has landed in a diversity of locations/contexts.

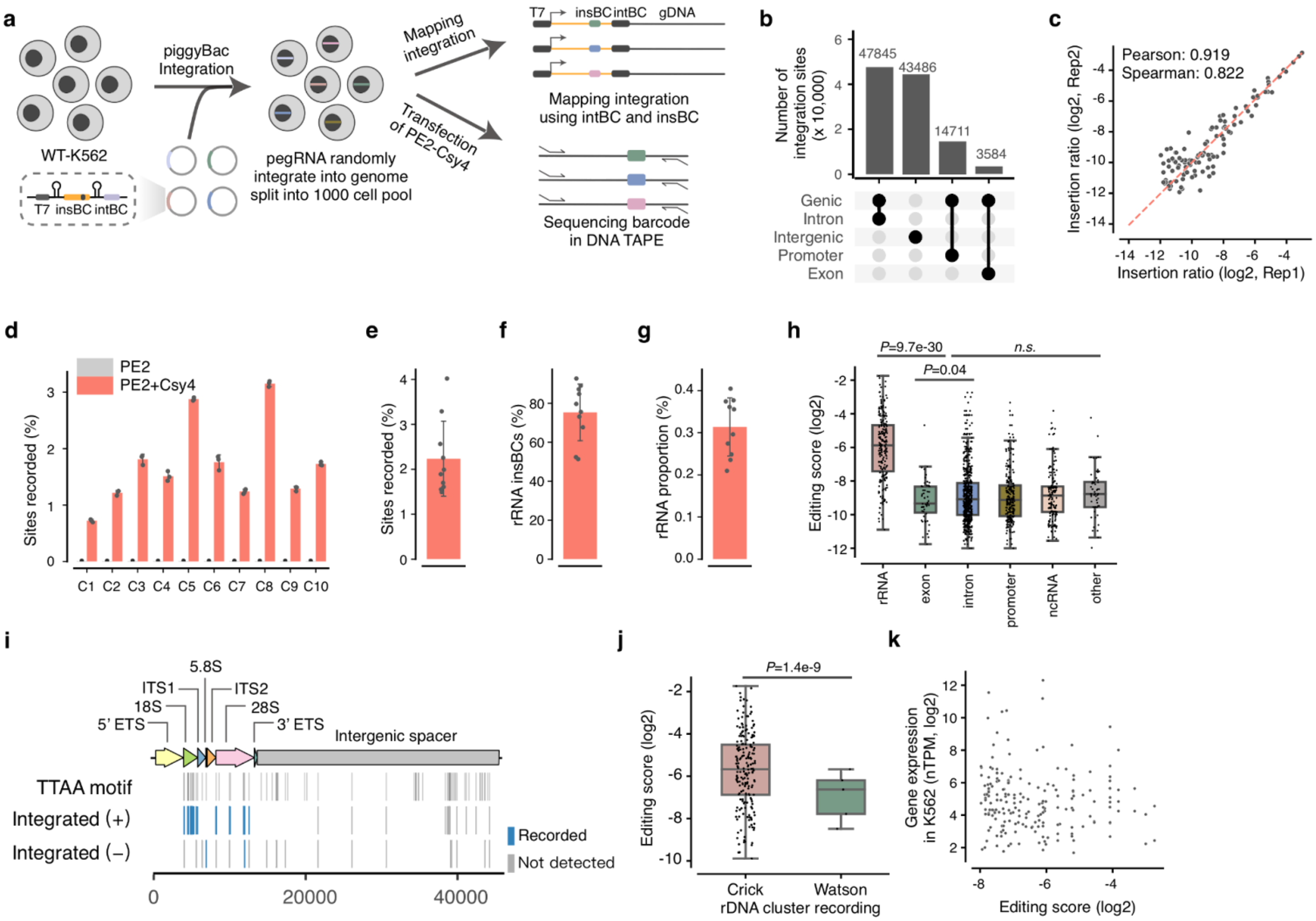

Supplementary Figure 2. Attempts to record endogenous transcription with ENGRAM. (a) Schematic of endogenous transcription recording. The recorder library with T7 promoter, pegRNA encoding 8bp insertion barcode (insBC), and 16bp location barcode (locBC) was integrated into K562 cells using piggyBac. Cells were split into 10 pools of 1000 cells/pool. After expansion, half of the cells were used to map integration sites, and the other half was used to record endogenous transcription activities. For mapping, genomic DNA is harvested and the genomic DNA is transcribed along with insBC and locBC using *in vitro* transcription. Integration sites were mapped based on a unique combination of insBCs and locBCs. For recording, PE2-Csy4 was transiently transfected in triplicates, and cells were harvested 3 days post-transfection. **(b)** Upset plot summarizing the integration site distribution, with 60.3% of the sites mapped to the genic region and 39.7% mapped to the intergenic region. **(c)** Endogenous gene expression recordings are reproducible across replicates. Two replicates of clone 5 were presented. Each dot represents a unique insBC. **(d)** Overall recording efficiency across 10 pools. Recording is only detectable when both PE2 and Csy4 are present. **(e)** 2.0% of the integrations are recorded (median, n=10). **(f)** The abundance of barcodes associated with rRNA transcripts. 77.4% of the recovered insBCs are associated with rRNA. **(g)** The proportion of integration sites mapped to ribosomal RNA clusters. 0.33% of the sites contribute to 77.4% of the recording, representing a ~238-fold enrichment. **(h)** Editing score by transcription category. Recording events were split into 6 categories based on their integration location. insBC associated with rRNA is much more abundant than other categories. P-values were obtained using the two-tailed Welch's t-test. **(i)** Integration of ENGRAM recorders into rDNA clusters. The vast majority of the active pegRNAs ("recorded") were oriented concordantly ("+") with the rDNA transcriptional unit (5'ETS to 3'ETS). **(j)** Boxplot showing the editing score (log2 ratio of barcode proportion) of pegRNAs integrated in a manner concordant ("+") vs. non-concordant ("-") with the orientation of the rDNA transcriptional unit. P-values were obtained using the two-tailed Welch's t-test. **(k)** Endogenous transcription recording is poorly correlated with gene expression in K562 cells, suggesting potential effects from factors such as gene functions and intron stability.

References

1. Anzalone, A. V. *et al.* Search-and-replace genome editing without double-strand breaks or donor DNA. *Nature* **576**, 149–157 (2019).
2. Muerdter, F. *et al.* Resolving systematic errors in widely used enhancer activity assays in human cells. *Nat. Methods* **15**, 141–149 (2018).
3. Nelson, J. W. *et al.* Engineered pegRNAs improve prime editing efficiency. *Nat. Biotechnol.* **40**, 402–410 (2022).
4. Chen, P. J. *et al.* Enhanced prime editing systems by manipulating cellular determinants of editing outcomes. *Cell* **184**, 5635–5652.e29 (2021).
5. Knapp, D. J. H. F. *et al.* Decoupling tRNA promoter and processing activities enables specific Pol-II Cas9 guide RNA expression. *Nat. Commun.* **10**, 1490 (2019).
6. Klein, J. C. *et al.* A systematic evaluation of the design and context dependencies of massively parallel reporter assays. *Nat. Methods* **17**, 1083–1091 (2020).
7. Djebali, S. *et al.* Landscape of transcription in human cells. *Nature* **489**, 101–108 (2012).
8. Li, X. *et al.* Chromatin context-dependent regulation and epigenetic manipulation of prime editing. *Cell* **187**, 2411–2427.e25 (2024).
