# Supplementary material for: Symbolic recording of signalling and cis-regulatory element activity to DNA
Source: Nature. 2024 Jul 17;632(8027):1073–81. doi: 10.1038/s41586-024-07706-4 (PMC11357993; doi:10.1038/s41586-024-07706-4)
Supplement: Supplementary file 2 — Reporting Summary [file 41586_2024_7706_MOESM2_ESM.pdf]

nature portfolio

Corresponding author(s): Jay Shendure

Last updated by author(s): May 17, 2024

Reporting Summary

Nature Portfolio wishes to improve the reproducibility of the work that we publish. This form provides structure for consistency and transparency in reporting. For further information on Nature Portfolio policies, see our Editorial Policies and the Editorial Policy Checklist.

Statistics

For all statistical analyses, confirm that the following items are present in the figure legend, table legend, main text, or Methods section.

n/a	Confirmed	
☐	☒	The exact sample size (*n*) for each experimental group/condition, given as a discrete number and unit of measurement
☐	☒	A statement on whether measurements were taken from distinct samples or whether the same sample was measured repeatedly
☐	☒	The statistical test(s) used AND whether they are one- or two-sided<br>*Only common tests should be described solely by name; describe more complex techniques in the Methods section.*
☒	☐	A description of all covariates tested
☒	☐	A description of any assumptions or corrections, such as tests of normality and adjustment for multiple comparisons
☐	☒	A full description of the statistical parameters including central tendency (e.g. means) or other basic estimates (e.g. regression coefficient) AND variation (e.g. standard deviation) or associated estimates of uncertainty (e.g. confidence intervals)
☐	☒	For null hypothesis testing, the test statistic (e.g. *F*, *t*, *r*) with confidence intervals, effect sizes, degrees of freedom and *P* value noted<br>*Give P values as exact values whenever suitable.*
☒	☐	For Bayesian analysis, information on the choice of priors and Markov chain Monte Carlo settings
☒	☐	For hierarchical and complex designs, identification of the appropriate level for tests and full reporting of outcomes
☐	☒	Estimates of effect sizes (e.g. Cohen's *d*, Pearson's *r*), indicating how they were calculated

Our web collection on statistics for biologists contains articles on many of the points above.

Software and code

Policy information about availability of computer code

Data collection	Illumina Next generation sequencing platform (Nextseq 500, Nextseq 2000 and Miseq) are used for data collection.
Data analysis	We have used bcl2fastq(v2.20), bwa (v0.7.1), NUPACK (4.0.0.27), custom Python code (Python 3.10) and custom R code (R 4.1) for analysis. Custom code is available at https://github.com/shendurelab/ENGRAM

For manuscripts utilizing custom algorithms or software that are central to the research but not yet described in published literature, software must be made available to editors and reviewers. We strongly encourage code deposition in a community repository (e.g. GitHub). See the Nature Portfolio guidelines for submitting code & software for further information.

Data

Policy information about availability of data

All manuscripts must include a data availability statement. This statement should provide the following information, where applicable:

- Accession codes, unique identifiers, or web links for publicly available datasets
- A description of any restrictions on data availability
- For clinical datasets or third party data, please ensure that the statement adheres to our policy

Raw sequencing data have been uploaded on Sequencing Read Archive (SRA) with associated BioProject ID PRJNA780310. Processed data is available at Github: https://github.com/shendurelab/ENGRAM. With the provided Jupyter notebook, the results and figures in the manuscript are fully reproducible. Plasmids for ENGRAM recorders (piggyBac-5’-ENGRAM and piggyBac-3’-FT-ENGRAM) have been deposited to Addgene (ID 179157 and ID 179158).
TF motif data was obtained from JASPAR database (https://jaspar2020.genereg.net/)

Field-specific reporting

Please select the one below that is the best fit for your research. If you are not sure, read the appropriate sections before making your selection.

☒ Life sciences ☐ Behavioural & social sciences ☐ Ecological, evolutionary & environmental sciences

For a reference copy of the document with all sections, see nature.com/documents/nr-reporting-summary-flat.pdf

Life sciences study design

All studies must disclose on these points even when the disclosure is negative.

Sample size	No sample size estimation was performed. In all experiments, we have performed 2-3 technical replicates for genome editing efficiency, which is sufficient to estimate the technical variation. Values measured in technical replications were used to calculate mean and standard deviations.
Data exclusions	No data was excluded
Replication	All experiments were performed at least with three replicates (transfection replicates, culture in separate wells) All attempts at replication were successful
Randomization	Randomization is not relevant to this study, as we only used human and mouse cells for measuring genome editing efficiencies.
Blinding	Blinding is not relevant to this study, as the values we measured in sequencing are quantitative and doesn't need subject judgment.

Reporting for specific materials, systems and methods

We require information from authors about some types of materials, experimental systems and methods used in many studies. Here, indicate whether each material, system or method listed is relevant to your study. If you are not sure if a list item applies to your research, read the appropriate section before selecting a response.

Materials & experimental systems

n/a	Involved in the study	
☒	☐	Antibodies
☐	☒	Eukaryotic cell lines
☒	☐	Palaeontology and archaeology
☒	☐	Animals and other organisms
☒	☐	Human research participants
☒	☐	Clinical data
☒	☐	Dual use research of concern

Methods

n/a	Involved in the study	
☒	☐	ChIP-seq
☒	☐	Flow cytometry
☒	☐	MRI-based neuroimaging

Eukaryotic cell lines

Policy information about cell lines

Cell line source(s)	HEK293T (ATCC), K562 (ATCC), Mouse embryonic stem cells (mESC, E14TG2a, gift from Dr. Christian Schröter
Authentication	All cell lines were used as received without further authericcation.
Mycoplasma contamination	Cell lines were not detected for mycoplasma contamination
Commonly misidentified lines (See ICLAC register)	Commonly misidentified cell lines were not used in this study.
